# The Epidemics of the Middle Ages

**Published:** 1835-04-01

**Authors:** 


*The Epidemics of the Middle Ages.* From the German of I. F. C. HECKER, M.D., Professor at Frederick William's University at Berlin. Translated by B. G. BABINGTON, M.D., F.R.S. No. II. *The Dancing Mania.*—London, 1835. 12mo. pp. 206.

FEW persons who have studied the meaning of medical nomenclature, can have failed to be struck with the term *St. Vitus's Dance.* The contortions of this disease have no resemblance to dancing; and St. Vitus is a saint so little known in this country, that we recollect to have seen a quaint conjecture that *chorea Sancti Viti* was a corruption of *chorea invita.* This conjecture, however, is opposed to the excellent, though not infallible rule of Griesbach, that uncommon words are corrupted into common ones, and not *vice versâ.* Moreover, St. Vitus, though but little known here, appears to have been a person of some note. He was a Sicilian youth, who suffered martyrdom, with Modestus and Crescentia, in the reign of Diocletian, A.D. 303.

The origin of the term *dance* is seen clearly enough, by the light which history affords. Those originally seized with the epidemic chorea really did dance, and though, in the progress of centuries, the disease has gradually become so mild that the term is inapplicable, it is still retained by the natural tradition of nomenclature. We shall follow Dr. Hecker, who begins his work with an account of the epidemic of 1374, though, as it appears afterwards, there had been much earlier dancing plagues.

"St. John's Dance. The effects of the *Black Death* had not yet subsided, and the graves of millions of its victims were scarcely closed, when a strange delusion arose in Germany, which took possession of the minds of men, and, in spite of the divinity of our nature, hurried away body and soul into the magic circle of hellish superstition. It was a convulsion which in the most extraordinary manner infuriated the human frame, and excited the astonishment of contemporaries for more than two centuries, since which time it has never reappeared. It was called the dance of St. John or of St. Vitus, on account of the Bacchantic leaps by which it was characterized, and which gave to those affected, whilst performing their wild dance, and screaming and foaming with fury, all the appearance of persons possessed. It did not remain confined to particular localities, but was propagated by the sight of the sufferers, like a demoniacal epidemic, over the whole of Germany, and the neighbouring countries to the north-west, which were already prepared for its reception by the prevailing opinions of the times.

"So early as the year 1374, assemblages of men and women were seen at Aix la Chapelle who had come out of Germany, and who, united by one common delusion, exhibited to the public both in the streets and in the churches the following strange spectacle. They formed circles hand in hand, and appearing to have lost all control over their senses, continued dancing, regardless of the bystanders, for hours together, in wild delirium, until at length they fell to the ground in a state of exhaustion. They then complained of extreme oppression, and groaned as if in the agonies of death, until they were swathed in cloths bound tightly round their waists, on which they again recovered, and remained free from complaint until the next attack. This practice of swathing was resorted to on account of the tympany which followed these spasmodic ravings, but patients were frequently relieved in a less artificial manner, by thumping and trampling upon the parts affected. While dancing they neither saw nor heard, being insensible to external impressions through the senses, but were haunted by visions, their fancies conjuring up spirits whose names they shrieked out; and some of them afterwards asserted that they felt as if they had been immersed in a stream of blood, which obliged them to leap so high. Others, during the paroxysm saw the heavens open and the Saviour enthroned with the Virgin Mary, as indeed the religious notions of the age were strangely and variously reflected in their imaginations." (P. 1.)

In severer cases, the attack began with epileptic convulsions. The patients fell to the ground, sprang up foaming at the mouth, and then commenced their dance amid strange contortions.*

The malady spread, and in many towns of Belgium "the dancers appeared with garlands in their hair, and their waists girt with cloths, that they might, as soon as the paroxysm was over, receive immediate relief on the attack of the tympany. This bandage was, by the insertion of a stick, easily twisted tight: many, however, obtained more relief from kicks and blows, which they found numbers of persons ready to administer; for, wherever the dancers appeared, the people assembled in crowds, to gratify their curiosity with the frightful spectacle." (P. 6.)

Strasburg was visited by a similar epidemic, in 1418: an old chronicle says,

> Viel hundert fingen zu Strassburg an
> Zu tanzen und springen Frau und Mann,
> Am offnen Markt, Gassen und Strassen
> Tag und Nacht ihrer viel nicht assen,
> Bis ihn das Wüthen wieder gelag,
> St. Vits Tanz ward genannt die Plag.†

It is obvious that the disease was considered one of great importance.

"Many who were seized at the sight of those affected, excited attention at first by their confused and absurd behaviour, and then by their constantly following the swarms of dancers. These were seen day and night passing through the streets, accompanied by musicians playing on bagpipes, and by innumerable spectators attracted by curiosity, to which were added anxious parents and relations, who came to look after those among the misguided multi-

* The foaming or spitting is mentioned in a Belgian chronicle, in the following line:

> Gens impacata cadit, dudum cruciata salvat.

[illegible]ecker very properly reads salivat for salvat, but he should have emendated [illegible]e beginning of the line too; for we would not rashly think so ill of a fellow-[illegible]reature (not even of the author of an old Belgian chronicle,) as to suppose that he would make a hexameter beginning with Gens impacata cadit: it should either be impacta or pacata; we prefer the latter; pacata standing for lassata, worn-out and lulled by the dancing.

† At Strasburgh hundreds of folks began
To dance and leap, both maid and man;
In open market, lane, and street,
They tripped along, nor cared to eat,
Until their plague had ceased to fright us.
'Twas named the dance of holy Vitus.

tude who belonged to their respective families. Imposture and profligacy played their part in this city also, but the morbid delusion itself seems to have predominated. On this account religion could only bring provisional aid, and therefore the town-council benevolently took an interest in the afflicted. They divided them into separate parties, to each of which they appointed responsible superintendents to protect them from harm, and perhaps also to restrain their turbulence. They were thus conducted on foot and in carriages to the chapels of St. Vitus, near Zabern and Rotestein, where priests were in attendance to work upon their misguided minds by masses and other religious ceremonies. After divine worship was completed, they were led in solemn procession to the altar, where they made some small offering of alms, and where it is probable that many were, through the influence of devotion and the sanctity of the place, cured of this lamentable aberration. It is worthy of observation, at all events, that the dancing mania did not recommence at the altars of the saint, and that from him alone assistance was implored, and through his miraculous interposition a cure was expected, which was beyond the reach of human skill." (P. 13.)

In discussing the causes of these epidemics, Dr. Hecker says,

"When we observe, however, that the first dancers in Aix la Chapelle appeared in July with St. John's name in their mouths, the conjecture is probable that the wild revels of St. John's day, A.D. 1374, gave rise to this mental plague, which thenceforth has visited so many thousands with incurable aberration of mind, and disgusting distortions of body.

"This is rendered so much the more probable, because some months previously the districts in the neighbourhood of the Rhine and the Maine had met with great disasters. So early as February, both these rivers had overflowed their banks to a great extent; the walls of the town of Cologne, on the side next the Rhine, had fallen down, and a great many villages had been reduced to the utmost distress. To this was added the miserable condition of Western and Southern Germany. Neither law nor edict could suppress the incessant feuds of the Barons, and in Franconia especially, the ancient times of club law appeared to be revived. Security of property there was none; arbitrary will everywhere prevailed; corruption of morals and rude power rarely met with even a feeble opposition; whence it arose that the cruel, but lucrative, persecutions of the Jews, were in many places still practised through the whole of this century, with their wonted ferocity. Thus, throughout the western parts of Germany, and especially in the districts bordering on the Rhine, there was a wretched and oppressed populace; and if we take into consideration, that among their numerous bands many wandered about, whose consciences were tormented with the recollection of the crimes which they had committed during the prevalence of the black plague, we shall comprehend how their despair sought relief in the intoxication of an artificial delirium. There is hence good ground for supposing that the frantic celebration of the festival of St. John, A.D. 1374, only served to bring to a crisis, a malady which had been long impending; and if we would further inquire how a hitherto harmless usage, which, like many others, had but served to keep up superstition, could degenerate into so serious a disease, we must take into account the unusual excitement of men's minds, and the consequences of wretchedness and want. The bowels, which in many were debilitated by hunger and bad food, were precisely the parts which in most cases were attacked with excruciating pain, and the tympanitic state of the intestines, points out to the intelligent physician, an origin of the disorder which is well worth consideration." (P. 23.)

These are by no means the most ancient examples of the kind. A hundred children were seized with a similar affection in the year 1237; and, in 1278, two hundred fanatics danced upon the bridge over the Moselle at Utrecht, "and would not desist, until a priest passed who was carrying the Host to a person that was sick; upon which, as if in punishment of the crime, the bridge gave way, and they were all drowned." (P. 28.)

Still more ancient traces are to be found.

"A similar event also occurred so early as the year 1027, near the convent church of Kolbig, not far from Bernburg. According to an oft repeated tradition, eighteen peasants, some of whose names are still preserved, are said to have disturbed divine service on Christmas eve, by dancing and brawling in the churchyard, whereupon the priest, Ruprecht, inflicted a curse upon them, that they should dance and scream for a whole year without ceasing. This curse is stated to have been completely fulfilled, so that the unfortunate sufferers at length sunk knee deep into the earth, and remained the whole time without nourishment, until they were finally released by the intercession of two pious bishops. It is said, that upon this, they fell into a deep sleep, which lasted three days, and that four of them died; the rest continuing to suffer all their lives from a trembling of their limbs." (P. 28.)

In the beginning of the sixteenth century, the disease began to give way.

"About this time the St. Vitus's dance began to decline, so that milder forms of it appeared more frequently, while the severer cases became more rare; and even in these, some of the important symptoms gradually disappeared. Paracelsus makes no mention of the tympanites as occurring after the attacks, although it may occasionally have occurred; and Schenck von Graffenberg, a celebrated physician of the latter half of the sixteenth century, speaks of this disease as having been frequent only in the time of his forefathers; his descriptions however are applicable to the whole of that century, and to the close of the fifteenth. The St. Vitus's dance attacked people of all stations, especially those who led a sedentary life, such as shoemakers and tailors; but even the most robust peasants abandoned their labours in the fields, as if they were possessed by evil spirits; and thus those affected were seen assembling indiscriminately, from time to time, at certain appointed places, and unless prevented by the lookers on, continuing to dance without intermission, until their very last breath was expended. Their fury and extravagance of demeanour so completely deprived them of their senses, that many of them dashed their brains out against the walls and corners of buildings, or rushed headlong into rapid rivers, where they found a watery grave. Roaring and foaming as they were, the bystanders could only succeed in restraining them by placing benches and chairs in their way, so that their strength might be exhausted by the high leaps they were thus tempted to take. As soon as this was the case, they fell as it were lifeless to the ground, and, by very slow degrees, again recovered their strength. Many there were, who even with all this exertion, had not expended the violence of the tempest which raged within them, but awoke with newly revived powers, and again and again mixed with the crowd of dancers, until at length the violent excitement of their disordered nerves was allayed by the great involuntary exertion of their limbs; and the mental disorder was calmed by the extreme exhaustion of the body. Thus the attacks themselves were in these cases, as in their nature they are in all nervous complaints, necessary crises of an inward morbid condition, which was transferred from the sensorium to the nerves of motion, and at an earlier period to the abdominal plexus, where a deep seated derangement of the system was perceptible from the secretion of flatus in the intestines.

"The cure effected by these stormy attacks was in many cases so perfect, that some patients returned to the factory or the plough as if nothing had happened. Others, on the contrary, paid the penalty of their folly by so total a loss of power, that they could not regain their former health, even by the employment of the most strengthening remedies. Medical men were astonished to observe that women in an advanced stage of pregnancy were capable of going through an attack of the disease, without the slightest injury to their offspring, which they protected merely by a bandage passed round the waist. Cases of this kind were not unfrequent so late as Schenck's time. That patients should be violently affected by music, and their paroxysms brought on and increased by it, is natural with such nervous disorders; where deeper impressions are made through the ear, which is the most intellectual of all the organs, than through any of the other senses. On this account the magistrates hired musicians for the purpose of carrying the St. Vitus's dancers so much the quicker through the attacks, and directed that athletic men should be sent among them, in order to complete the exhaustion which had been often observed to produce a good effect. At the same time there was a prohibition against wearing red garments, because, at the sight of this colour, those affected became so furious, that they flew at the persons who wore it, and were so bent upon doing them an injury that they could with difficulty be restrained. They frequently tore their own clothes whilst in the paroxysm, and were guilty of other improprieties, so that the more opulent employed confidential attendants to accompany them, and to take care that they did no harm either to themselves or others. This extraordinary disease was, however, so greatly mitigated in Schenck's time, that the St. Vitus's dancers had long since ceased to stroll from town to town; and that physician, like Paracelsus, makes no mention of the tympanitic inflation of the bowels. Moreover, most of those affected, were only annually visited by attacks; and the occasion of them was so manifestly referrible to the prevailing notions of that period, that if the unqualified belief in the supernatural agency of saints could have been abolished, they would not have had any return of the complaint. Throughout the whole of June, prior to the festival of St. John, patients felt a disquietude and restlessness which they were unable to overcome. They were dejected, timid, and anxious; wandered about in an unsettled state, being tormented with twitching pains, which seized them suddenly in different parts, and eagerly expected the eve of St. John's day, in the confident hope, that by dancing at the altars of this saint, or of St. Vitus (for in the Breisgau aid was equally sought from both), they would be freed from all their sufferings. This hope was not disappointed; and they remained, for the rest of the year, exempt from any further attack, after having thus, by dancing and raving for three hours, satisfied an irresistible demand of nature. There were at that period two chapels in the Breisgau, visited by the St. Vitus's dancers; namely, the Chapel of St. Vitus at Biessen, near Breisach, and that of St. John, near Wasenweiler; and it is probable that in the south-west of Germany, the disease was still in existence in the seventeenth century." (P. 46.)

Dr. Hecker attributes the ultimate disappearance of the dancing plague to the thirty-years' war, which raged in Germany from 1618 to 1648; for, "although the unspeakable calamities which they brought upon Germany, both during their continuance and in their immediate consequences, were by no means favourable to the advance of knowledge, yet, with the vehemence of a purifying fire, they gradually effected the intellectual regeneration of the Germans: superstition, in her ancient form, never again appeared, and the belief in the dominion of spirits, which prevailed in the middle ages, lost for ever its once formidable power." (P. 51.)

In the second chapter, Dr. Hecker treats of the dancing mania in Italy, and more especially of *Tarantism*. This was the disease supposed to be occasioned by the bite of the Tarantula, a ground spider common in Apulia. The symptoms were as follows:

"Those who were bitten, generally fell into a state of melancholy, and appeared to be stupified, and scarcely in possession of their senses. This condition was, in many cases, united with so great a sensibility to music, that, at the very first tones of their favorite melodies, they sprang up, shouting for joy, and danced on without intermission, until they sunk to the ground exhausted and almost lifeless. In others, the disease did not take this cheerful turn. They wept constantly, and as if pining away with some unsatisfied desire, spent their days in the greatest misery and anxiety. Others, again, in morbid fits of love, cast their longing looks on women, and instances of death are recorded which are said to have occurred under a paroxysm of either laughing or weeping." (P. 65.)

The early accounts do not state that the patients felt an irresistible propensity to dancing, or were cured by it: this was the case, however, at a later period.

"At the close of the fifteenth century we find that Tarantism had spread beyond the boundaries of Apulia, and that the fear of being bitten by venomous spiders had increased. Nothing short of death itself was expected from the wound which these insects inflicted, and if those who were bitten escaped with their lives, they were said to be seen pining away in a desponding state of lassitude. Many became weak-sighted or hard of hearing, some lost the power of speech, and all were insensible to ordinary causes of excitement. Nothing but the flute or the cithern afforded them relief. At their sounds they awoke as it were by enchantment, opened their eyes, and moving slowly at first, according to the measure of the music, were, as the time quickened, gradually hurried on to the most passionate dance. It was generally observable that country people, who were rude, and ignorant of music, evinced on these occasions an unusual degree of grace, as if they had been well practised in elegant movements of the body; for it is a peculiarity in nervous disorders of this kind, that the organs of motion are in an altered condition, and are completely under the control of the overstrained spirits. Cities and villages alike resounded throughout the summer season with the notes of fifes, clarinets, and Turkish drums; and patients were every where to be met with who looked to dancing as their only remedy. Alexander ab Alexandro, who gives this account, saw a young man in a remote village who was seized with a violent attack of Tarantism. He listened with eagerness and a fixed stare to the sound of a drum, and his graceful movements gradually became more and more violent until his dancing was converted into a succession of frantic leaps, which required the utmost exertion of his whole strength. In the midst of this overstrained exertion of mind and body the music suddenly ceased, and he immediately fell powerless to the ground, where he lay senseless and motionless until its magical effect again aroused him to a renewal of his impassioned performances.

"At the period of which we are treating there was a general conviction, that by music and dancing the poison of the Tarantula was distributed over the whole body, and expelled through the skin, but that if there remained the slightest vestige of it in the vessels, this became a permanent germ of the disorder, so that the dancing fits might again and again be excited *ad infinitum* by music. This belief, which resembled the delusion of those insane persons, who, being by artful management freed from the imagined causes of their sufferings, are but for a short time released from their false notions, was not entertained without the most injurious effects: for in consequence of it those affected necessarily became by degrees convinced of the incurable nature of their disorder. They expected relief indeed, but not a cure, from music; and when the heat of summer awakened a recollection of the dances of the preceding year, they, like the St. Vitus's dancers of the same period before St. Vitus's day, again grew dejected and misanthropic, until, by music and dancing, they dispelled the melancholy which had become with them a kind of sensual enjoyment." (P. 75.)

The third chapter is on the *Tigretier*, the dancing mania of Abyssinia; and the fourth is on the Effects of Sympathy. A curious instance is given in it of an epidemic hysteria among the girls of a cotton mill, arising in the first case from fright and disgust, and in the others from imitation; together with an account of the strange effects of ecstasy and frenzy among the Convulsionaires, the Shakers, Jumpers, and other enthusiastic religionists.

The Appendix contains several interesting pieces, including extracts from old German chronicles. There are also eight pieces of music, to which the persons bitten by the tarantula danced: one is called the *primus modus Tarentella*, another the *tono hypodorio*, and so on. The music to which the Tarantati danced, and of which Kircher has preserved these specimens, will not, to modern ears, convey impressions so delightful and exhilarating as it appears to have produced at the period when that singular malady prevailed. Indeed, the invariable termination in a minor key, seems to prove that, amid all their wild excitement, melancholy was the dominant feeling of their minds.

It would now be our duty to give a general opinion on the merits of Dr. Hecker's work; but the translator, Dr. Babington, has given so good a review in his preface, that we cannot do better than extract a considerable part of it: for nothing can be more agreeable than to find our ideas anticipated, in language far more lucid than any in which we could have hoped to embody them.

"The mind and the body reciprocally and mysteriously affect each other, and the maladies which are the subject of these pages, are so intimately connected with the disordered state of both, that it is often difficult to determine on which they more essentially depend, or which they more seriously influence.

"The physician will probably be led by their contemplation to admit that the imagination has a larger share in the production of disease than he might, without a knowledge of the striking facts here recorded, have supposed to be within the limits of possibility. He has, no doubt, already observed, that joy will affect the circulation, grief the digestion, that anger will heat the frame as perniciously as ardent spirits, and that fear will chill it as certainly as ice; but he may not have carried his observation to the extent of perceiving, that not only single and transient effects, but specific diseases are produced through the agency of mental impressions, and he may therefore still be surprised to find that the dances of St. John and of St. Vitus, as they formerly spread by sympathy from city to city, gave rise to the same deviations from bodily health, in all the individuals whom they attacked; that Tarantism was the same disease, whether medically or morally considered, all over Italy; and that the 'Lycanthropia' of the past, and the 'Leaping Ague' of the present times, have each its respective train of peculiar symptoms." (*Pref.* vii.)

"I thus venture to hope, that by bestowing a leisure hour on this small portion of medical history, the physician may enlarge his knowledge of disease, and the moralist may gather a hint for the intellectual improvement of his fellow-men. The author has, however, a more extended object in view; the histories of particular epidemics are with him but the data from which we are to deduce the general laws that govern human health in the aggregate. Whether there be such an *entity* as collective organic life, and whether, as a consequence, there exist general laws which regulate its healthy or morbid condition, I do not here undertake to determine; but the notion is peculiar, and in order that it may be more fully exposed to the reader, I have translated, as an introduction to the present volume, an Appeal which Dr. Hecker has made to the medical profession of his own country for assistance in his undertaking. If, in the course of the remarks contained in this address, he has been somewhat severe in his censure of the neglect, both in this country and in France, of the study of Medical History, I freely confess myself to be one of those who are more anxious to profit by his castigation than to dispute its justice." (*Pref.* xii.)

